# Comprehensive Analysis of Prognostic Alternative Splicing Signatures in Endometrial Cancer

**DOI:** 10.3389/fgene.2020.00456

**Published:** 2020-05-29

**Authors:** Peigen Chen, Junxian He, Huixia Ye, Senwei Jiang, Yunhui Li, Xiaomao Li, Jing Wan

**Affiliations:** Department of Gynecology, The Third Affiliated Hospital of Sun Yat-Sen University, Guangzhou, China

**Keywords:** endometrial cancer, alternative splicing events, TCGA, precision medicine, prognostic signature, splicing factors

## Abstract

**Background:**

Alternative splicing (AS) is one of the critical post-transcriptional regulatory mechanisms of various cancers and also plays a crucial role in the development of cancers, including endometrial cancer (EC).

**Methods:**

The splicing data and gene expression profiles of EC were obtained from The Cancer Genome Atlas. The corresponding clinical data were extracted from TCGA-CDR. With univariate Cox regression analysis, least absolute shrinkage and selection operator model, and multivariate Cox regression analysis, the survival-related AS events were selected. Functional enrichment analysis was also performed to investigate the functions of these AS events. Splicing factors and AS regulation network were constructed to understand the correlation among these AS events.

**Result:**

A total of 1826 AS events were identified as survival-related events. Functional enrichment analysis showed that these AS events were associated with several immune system-related processes. Then, the prognostic signatures were developed based on these survival-related events and acted as an independent prognostic factor for EC. Splicing factors and AS regulation network were also constructed to understand the regulatory mechanisms of AS events in EC.

**Conclusion:**

This study systematically analyzed the role of AS events in EC and developed the prognostic model for EC.

## Introduction

Endometrial cancer (EC) is the most common gynecologic malignancy and also the fourth most common cancer (about 4.8% of all cancers) in women (Ferlay et al., 2015). EC is generally divided into two subtypes, estrogen-dependent subtype (type I), and estrogen-independent subtype (type II). Type I EC, commonly referred to as the endometrioid type, comprises about 80% of all EC. Type II EC, more common in elder patients, is more aggressive and accounts for at least 40% of EC-related deaths (Bokhman, 1983; Jia et al., 2014; Matias-Guiu and Davidson, 2014; Zheng et al., 2018). According to cancer statistics in China, the incidence rate of EC was 634 per 100,000 and the mortality rate was 21.8 per 100,000 in 2017. The incidence rate of EC was 4 to 20 times higher in patients aged 50 or older than in patients under 50 (Chen et al., 2016). Although most of the cases of EC are diagnosed in the early stage with relatively good prognosis, there are still over 20% patients who die from the disease due to distant metastasis and recurrence, which often lead to poor response to conventional therapy. Therefore, it is essential to screen for biomarkers to predict metastasis and recurrence of EC and monitor the prognosis of EC patients effectively.

Alternative splicing (AS) is one of the critical post-transcriptional regulation mechanisms, which is one of the reasons for the diversity of the transcriptome and proteome. A previous study shows that about 95% multi-exon human genes are the products of AS events (Pan et al., 2008). As events are usually divided into seven categories, including Alternate Acceptor site (AA), Alternate Donor site (AD), Alternate Promoter (AP), Alternate Terminator (AT), Exon Skip (ES), Retained Intron (RI), and Mutually Exclusive Exons (ME). AS can generate alternative mRNA transcripts and encode a series of protein isoforms that differ in structure and function (Stamm et al., 2005). Due to functional importance and high frequency of occurrence, changes in AS often affect the homeostasis of cells, which may be related to cancers. Some cancers can use AS to produce proteins that are conducive to the proliferation and invasion of cancer cells (Dargahi et al., 2014). Emerging studies suggest that abnormal AS events are closely related to the development of cancers (Oltean and Bates, 2014; Climente-Gonzalez et al., 2017; Singh and Eyras, 2017; Zong et al., 2018; Yang et al., 2019) and some AS events are targets of prognosis and treatment (Pajares et al., 2007; Venables et al., 2008; Griffith et al., 2013).

To determine whether aberrant AS events have clinical significance for EC patients, we obtained RNA-seq data from TCGA (The Cancer Genome Atlas) program, AS data from TCGA SpliceSeq, and clinical data from TCGA-CDR (Liu et al., 2018). Then, we systematically studied the relationship between aberrant AS events and prognosis of EC patients. The purpose of this study is to investigate whether AS events are associated to the overall survival (OS) of EC patients and try to find out some novel and appropriate targets for treatments of EC patients.

## Materials and Methods

We used R software (version 3.5.1) (R Core Team, 2018) and Bioconductor (Gentleman et al., 2004) for all statistical analyses in our whole study.

### Data Acquisition and Preprocessing

RNA sequencing expression data normalized by Fragments Per Kilobase of transcript per Million mapped reads (FPKM) were obtained from the University of California, Santa Cruz Genome Browser (UCSC: version 2017–09–14)^1^, a public database that contains the genome and its information. The corresponding clinical data were extracted from TCGA-CDR (TCGA Pan-cancer Clinical Data Resource) dataset (Liu et al., 2018). TCGA-CDR was an authoritative clinical dataset built for analyzing clinical pathology annotations of more than 11,000 cancer patients in the TCGA program. Patients whose OS time was less than 30 days were excluded from our study, because these patients may have died or quit due to non-tumor factors. Totally, 508 patients were included in our study.

Percent Spliced In (PSI) values of AS events were obtained from TCGA SpliceSeq (version 2018–11–25)^2^ (Ryan et al., 2016). The data portal provided a comprehensive data profile of seven types of AS events that related to 33 cancer types and applied the PSI to identify its quantification. The selection criteria of AS events included the following: (1) the average PSI values >0.05 and (2) the standard deviation of all AS event’s values >0.01. Then, “impute” R package was used to replace the “null” data by imputing for microarray data (Hastie et al., 2020).

### Survival Analysis and Development of Prognostic Signatures

Univariate Cox regression performed by “survival” R package (Terry and Therneau, 2000) and LASSO (least absolute shrinkage and selection operator) regression Cox analysis performed by “glmnet” R package with number of lambda = 1000 (Friedman et al., 2010) were used to screen the prognostic-related AS events with *p*-value < 0.05. Clinical outcome and gene expression profiles were input in LASSO algorithm. Lambda.min is the cutoff point that brings minimum mean cross-validated error. Those with the highest lambda value were selected as based on the lambda.min to selected significant predictive predictors for further analysis. We then visualized the genes selected by univariate Cox regression analysis by bar plot drawn by Prism 7.0 and upset plot drawn by “UpSetR” R package (Conway et al., 2017).

Multivariate Cox regression analysis performed by “survival” R package was subsequently used to develop prognostic signature in each AS type and further evaluated the prognostic value of each AS events in EC. The risk score of each prognostic signature was then calculated according to the formula: risk score = $\sum_{i=1}^{n}\beta \,i*x\,i$ (β stands for the regression coefficient) (Yang et al., 2011). Receiver operating characteristic (ROC) curve and area under the curve (AUC) were calculated by “survivalROC” R package (Saha-Chaudhuri, 2013) to estimate the predictive accuracy of each signature. EC patients were divided into a high-risk group and a low-risk group according to the risk score. Kaplan–Meier (KM) survival curves were then used to compare the survival differences between high-risk group and low-risk group.

Risk score models and some following important clinical features: grade, stage, pathology (including endometrioid endometrial adenocarcinoma, serous endometrial adenocarcinoma, and mixed serous and endometrioid endometrial adenocarcinoma), age, and BMI were integrated into univariate and multivariate Cox regression analysis to evaluate these features as the independent risk factors.

### Functional Enrichment Analysis

Metascape^3^ is an online functional enrichment tool that included abundant functional annotation such as KEGG Pathway, Reactome Pathway, Canonical Pathway, GO Biological Processes, and CORUM (The comprehensive resource of mammalian protein complexes) (Zhou et al., 2019). Functional enrichment analysis and visualization were performed by Metascape (see text footnote 3) based on the corresponding genes of prognostic-related AS events with *p*-value < 0.001 as cutoff value. Those terms selected with *p*-value < 0.01 and the numbers of genes higher or equal to 3 were considered as significant terms.

For understanding the interactions between protein and protein, the protein-to-protein (PPI) network was also established by Metascape based on BioGrid database (Stark et al., 2006), InWeb_IM database (Li et al., 2017), and OmniPath database. The Molecular Complex Detection (MCODE) algorithm (Bader and Hogue, 2003) was then used to identify the modules of the PPI network according to the following filter: degree cutoff = 2, node score cutoff = 0.2, k-core = 2, and maximum depth = 10.

### Splicing Factor and AS Regulatory Network Construction

To analyze the correlation between survival-associated AS events and splicing factors, we then constructed a splicing factor and AS regulatory network. A total of 404 splicing factors (SF) were obtained from a study by Seiler et al. (2018). The expression data of SF were extracted from TCGA database. Then, Pearson correlation test was applied to evaluate the potential relevance of SF and the survival-associated AS events with *p*-value < 0.05 and correlation coefficient >0.3. The regulatory network was then visualized by Cytoscape (version 3.7.0).

## Results

### Data Acquisition and Preprocessing

**FIGURE 1:**
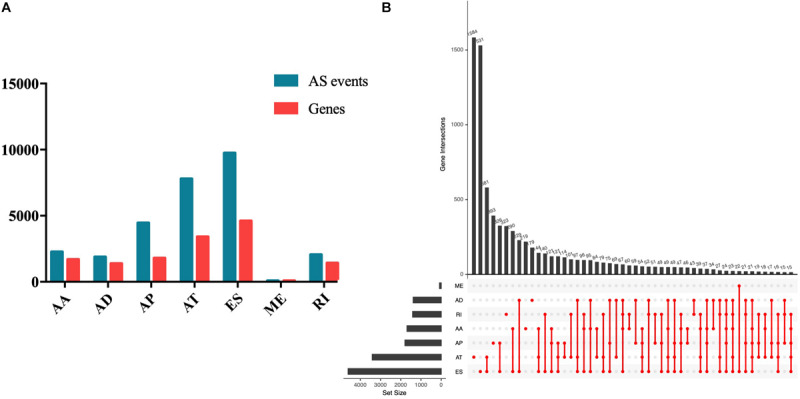
Summary of AS events of endometrial carcinoma. **(A)** Counts of AS events and correlation genes. **(B)** Counts of survival-associated AS events and correlation genes.

Seven types of AS events were involved in the study, including AA, AD, AP, AT, ES, RI, and ME. In total, 28,282 AS events of 8140 genes in 508 EC patients were obtained, including 2270 AS events of 1691 genes, 1877 AD events of 1386 genes, 4458 AP events of 1792 genes, 7796 AT events of 3411 genes, 86 ME events of 85 genes, and 2051 RI events of 1413 genes (Figure 1A). As the Upset plot in Figure 1B showed, one gene could undergo up to four types of AS events.

### Selection of Survival Associated With AS Events

**FIGURE 2:**
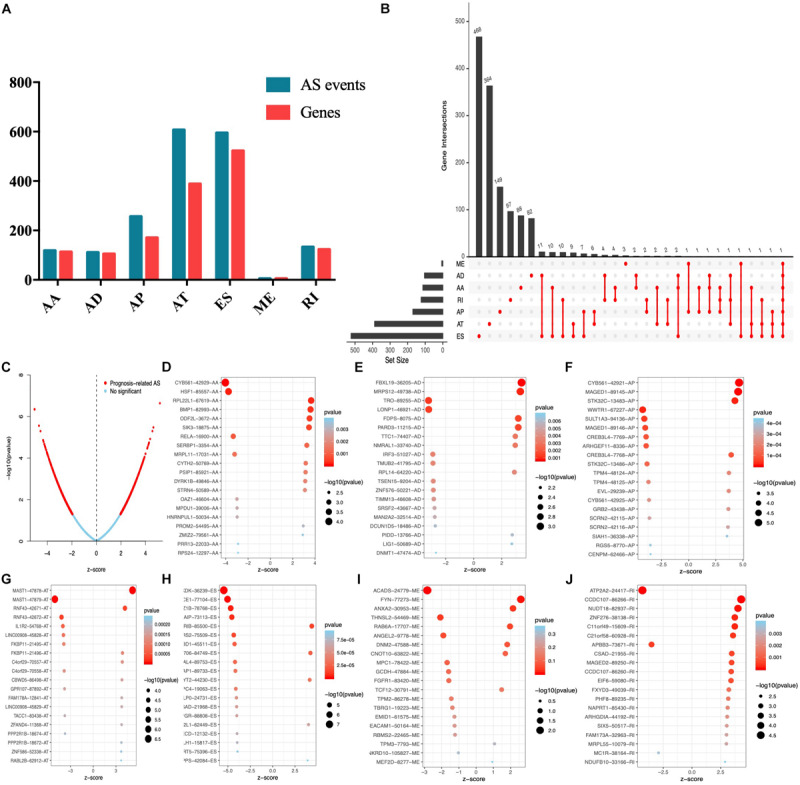
Top 20 significant AS events of endometrial carcinoma. **(A)** The UpSet intersection diagram of seven types of AS events. **(B)** The UpSet intersection diagram of survival-associated AS events. **(C)** The volcano plot of survival-related AS events (red dots). The blue dots indicate AS events that aren’t related to survival. **(B–H)** The top 20 survival-related AS events based on AA **(D)**, AD **(E)**, AP **(F)**, AT **(G)**, ES **(H)**, ME **(I)**, and RI **(J)**.

Univariate Cox analysis was performed to select the survival associated with AS events. In total, 1826 AS events significantly associated with OS were selected (Supplementary Table S1) and the number of each AS event is listed in Figure 2A. We also found that up to four survival-associated events could occur on the same gene (Figure 2B). According to the volcano map in Figure 2C, we found that the occurrence of AS events was significantly associated with patients’ survival. The top 20 most significantly associated survival events of each AS event type are shown in Figures 2D–J.

### Functional Enrichment Analysis of Survival Associated With AS Events

**FIGURE 3:**
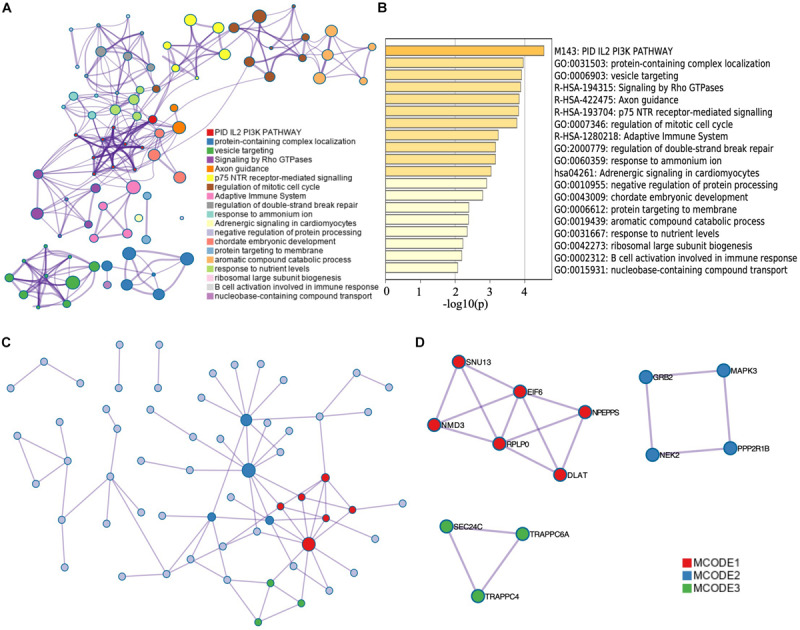
Functional enrichment analysis of corresponding genes of survival-related AS events. The network **(A)** and bar plot **(B)** of enrich terms of correlation genes of survival-related AS events. The dot in **(A)** represents every corresponding genes of top significant survival-related AS events. The depth of the color of the bar plot **(B)** indicates the significance of *p*-value. Those enrichment terms including more node were more significant. **(C)** Protein–protein interaction network of top significant survival-related AS events. **(D)** Top MCODE of the PPI network.

The corresponding top genes of prognostic-related AS events (*p* < 0.001) were input into Metascape to investigate the pathways and biological functions. As the results showed, these genes mainly enriched in PID IL2 PI3K pathway (M143) and Adrenergic signaling in cardiomyocytes pathway (hsa04261). The biological processes that the genes mainly clustered in included adaptive immune system (R-HSA-1280218), regulation of mitotic cell cycle (GO:0007346), and axon guidance (R-HSA-422475) (*p* < 0.01) (Figure 3A). Figure 3B illustrated the interaction between the pathways and biological functions terms.

Besides, we constructed a PPI network by Metascape (Figure 3C). Modules of the PPI network were then identified by Molecular Complex Detection (MCODE) (Figure 3D). Functional enrichment analysis, including pathway and biological process, was also applied to each module selected by MCODE (Table 1). We found that MCODE 1 mainly enriched in ribosome biogenesis (GO:0042254) and MCODE 2 mainly enriched in signaling by NTRK1 (TRKA). We also found that MCODE 3 mainly enriched in COPII vesicle coating, vesicle targeting, rough ER to cis-Golgi, and COPII-mediated vesicle transport.

**TABLE 1:** Functional enrichment terms of the key modules of the PPI network.

**MCODE**	**GO**	**Description**	**Log10 (*P*)**
MCODE_1	GO:0042254	Ribosome biogenesis	–6.5
MCODE_1	GO:0042273	Ribosomal large subunit biogenesis	–6.3
MCODE_1	hsa03008	Ribosome biogenesis in eukaryotes	–5.8
MCODE_2	R-HSA-187037	Signaling by NTRK1 (TRKA)	–6.9
MCODE_2	R-HSA-166520	Signaling by NTRKs	–6.6
MCODE_2	R-HSA-6811558	PI5P, PP2A, and IER3 Regulate PI3K/AKT Signaling	–6.5
MCODE_3	GO:0048208	COPII vesicle coating	–7.7
MCODE_3	GO:0048207	Vesicle targeting, rough ER to cis-Golgi	–7.7
MCODE_3	R-HSA-204005	COPII-mediated vesicle transport	–7.7

### Prognostic Signatures Selecting and Survival Analysis

**FIGURE 4:**
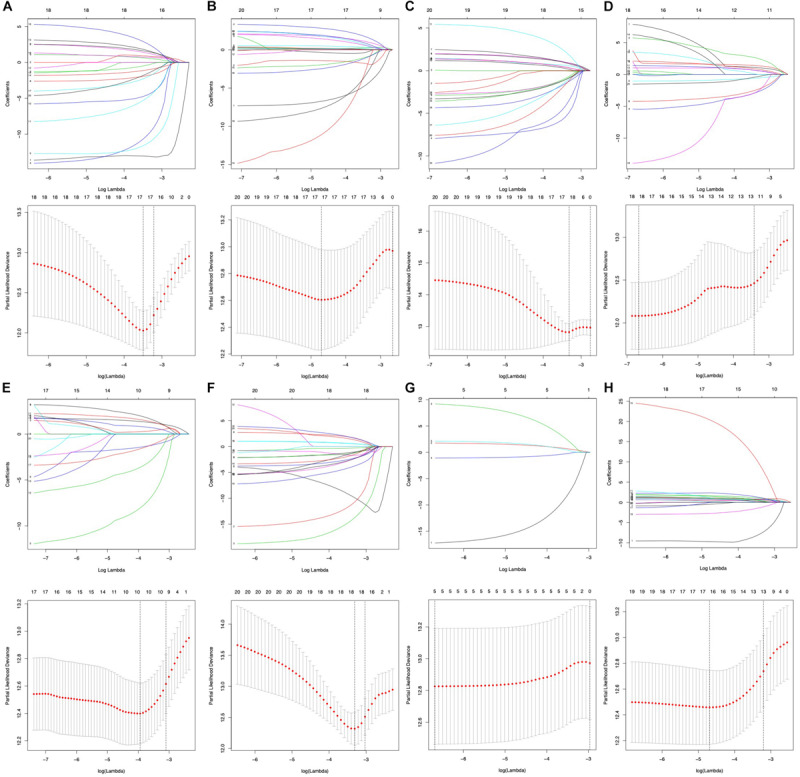
Prognostic signatures of survival-related AS events constructed based on LASSO COX analysis. **(A–H)** Represent the result of AA, AD, AP, AT, ES, ME, RI, and all seven types of AS events.

To select key prognostic signatures accurately, LASSO algorithm was then performed to develop prognostic signatures according to seven types of AS events [*n*(AA) = 17, *n*(AP) = 19, *n*(AT) = 10, *n*(AD) = 18, *n*(ME) = 5, *n*(ES) = 18 and *n*(RI) = 16] following the univariate Cox analysis (Figures 4A–H and Supplementary Table S2). Multivariate Cox analysis was then used to construct predictive models based on the AS events that LASSO algorithm selected. The prognostic signature of the entire seven types of AS events is listed in Table 2.

**TABLE 2:** Prognostic signatures of AS events of EC.

**Type**	**Formula**	**Hazard ratio (95% CI)**	**AUC**
AA	CYB561|42929|AA*-7.617147 + BMP1|82993|AA*3.577351 + SIK3|18875|AA *2.408505 + CYTH2|50769|AA*2.958247 + OAZ1|46604|AA*-21.098957 + HNRN PUL1|50034|AA*-3.146763 + PROM2|54495|AA*2.713899 + ZMIZ2|79561|AA *2.080869 + PRR13|22033|AA*-8.792135	1.115 (1.037–1.199)	0.750
AD	FBXL19|36205|AD*2.268930 + LONP1|46921|AD*-9.375422 + PARD3|11215| AD*1.7639 + TTC1|74407|AD*2.453769 + NMRAL1|33740|AD*1.211921 + IRF3|51027|AD*-2.751666 + RPL14|64220|AD*5.437216 + TSEN15|9204|AD*-3.3160 86 + ZNF576|50221|AD*-3.695564 + SRSF2|43667|AD*-3.391570 + MAN2A2 |32514|AD*-4.747463 + DCUN1D5|18486|AD*-6.899028 + PIDD|13766|AD* 1.645522 + DNMT1|47474|AD*-7.984222	1.068 (0.998–1.143)	0.781
AP	CYB561|42921|AP*8.928847 + MAGED1|89145|AP*1.884183 + STK32C|13483|AP*5.1870 96 + WWTR1|67227|AP*-5.635166 + ARHGEF11|8336|AP*-3.997173 TPM4|48124|AP*1.635788 + EVL|29239|AP*0.828410 + CYB561|42925|AP*7.034754 + GR B2|43438|AP*2.300765 + SIAH1|36338|AP*3.623569 + RGS5|8770|AP*- 15.639138 + CENPM|62466|AP*-1.410251	1.043 (1.019–1.067)	0.802
AT	MAST1|47878|AT*1.767971 + IL1R2|54768|AT*-10.532854 + LINC00908|4582 8|AT*-2.199804 + C4orf29|70557|AT*3.125794 + CBWD5|86498|AT*2.144659 + GPR107|87892|AT*-5.922088 + PPP2R1B|18674|AT*-2.645103	1.067 (0.999–1.139)	0.752
ES	HACE1|77104|ES*-15.189826 + CCZ1B|78768|ES*-20.165353 + SNCAIP|7311 3|ES*-4.298498 + SCRIB|85500|ES*1.292943 + MBD1|45511|ES*2.119335 + ZNF 706|84749|ES*2.490369 + NGFRAP1|89733|ES*-8.806658 + PCYT2|44230|ES* 1.147334 + RPLP0|24731|ES*-5.995624 + CSAD|21968|ES*-3.361527 + NHP2L1 |62449|ES*3.502868 + FOLH1|15817|ES*-5.784057 + SIRT5|75396|ES*-5.121171 + NPEPPS|42084|ES*2.674696	1.016 (1.002–1.030	0.866
ME	ACADS|24779|ME*-19.062553 + FYN|77273|ME*1.852911 + ANXA2|30953| ME*10.784103 + RAB6A|17707|ME*2.198519	1.127 (1.054–1.204)	0.566
RI	ATP2A2|24417|RI*-11.719351 + NUDT18|82937|RI*2.151315 + ZNF276|38138| RI*1.537001 + C11orf49|15609|RI*1.365296 + CSAD|21955|RI*1.236415+ MAGED2|89250|RI*2.626241 + CCDC107|86260|RI*2.888269 + ARHGDIA|44192|RI*27.7 92332 + MC1R|38164|RI*-3.219843	1.031 (1.013–1.048)	0.762
ALL	BCKDK|36239|ES*-17.454109 + MAST1|47878|AT*1.331098 + HACE1|77104| ES*-14.724703 + CCZ1B|78768|ES*-15.302180 + CYB561|42921|AP*2.428443+ MAGED1|89145|AP*3.379968 + SNCAIP|73113|ES*-3.874507 + ATP2A2|24417| RI*-7.165311 + STK32C|13483|AP*3.374097 + MBD1|45511|ES*-1.765549+ ZNF706|84749|ES*5.359007 + TCEAL4|89753|ES*-4.954079	1.010 (1.006–1.015)	0.798

*Means the last two letters stand for the type of AS events.

**FIGURE 5:**
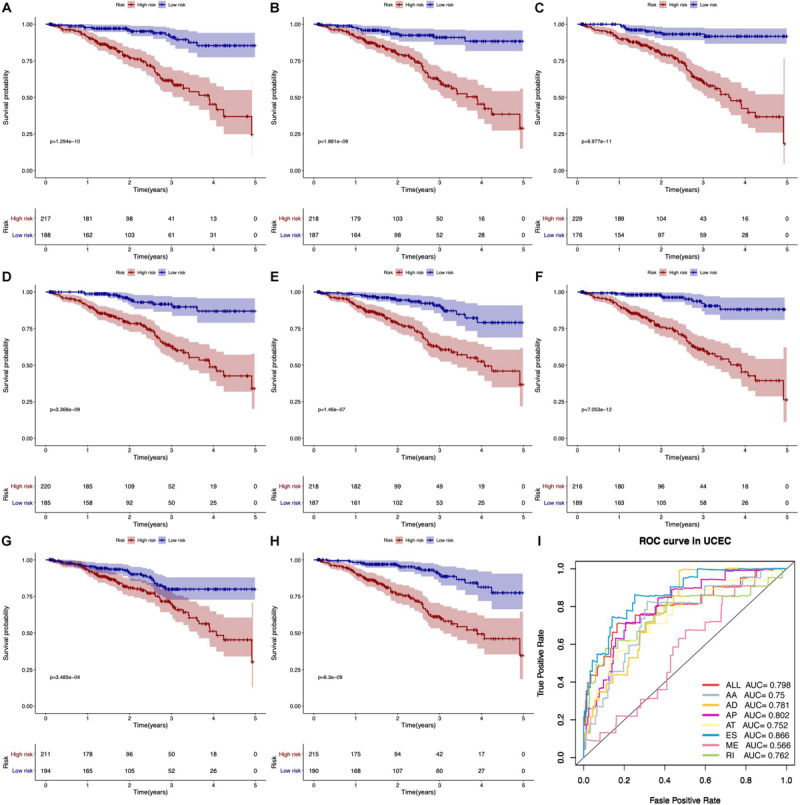
Kaplan–Meier and ROC curves of prognostic predictors. **(A–G)** The Kaplan–Meier curves indicate the survival probability of high-risk group patients (red line) and low-risk group patients (blue line) based on seven types of AS events, respectively. **(H)** The Kaplan–Meier curves represent the survival probability of high- and low-risk group patients based on all seven types. **(I)** The ROC curves of all prognostic predictors.

According to KM survival analysis, we found that seven types of AS prognostic signatures were significantly associated with OS time of EC patients (Figures 5A–H). Then, the ROC curve verified the predictive performance of these prognostic signatures (Figure 5I). Most AUC values of AS prognostic signatures were higher than 0.7. It meant that these AS prognostic signatures had satisfactory prediction accuracy.

**FIGURE 6:**
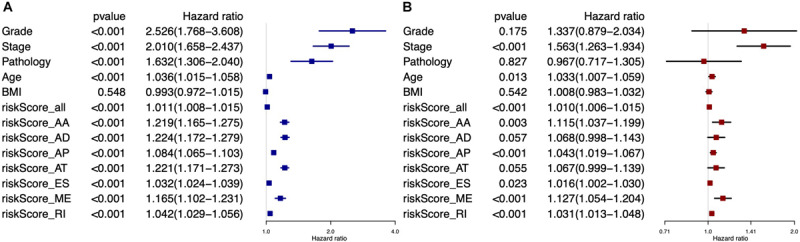
Forest plots for all prognostic predictors and clinical features based on univariate Cox analysis **(A)** and multivariate Cox analysis **(B)**.

Univariate Cox analysis (Figure 6A) and multivariate Cox analysis (Figure 6B) of clinical factors and risk score model showed that stage (HR = 1.563, 95% CI: 1.263–1.934, *p* < 0.001), age (HR = 1.033, 95% CI: 1.007–1.059, *p* = 0.013), risk score model of all types of AS events (HR = 1.010, 95% CI: 1.006–1.015, *p* < 0.001), risk score model of AA (HR = 1.115, 95% CI: 1.037–1.199, *p* = 0.003), AP (HR = 1.043, 95% CI: 1.019–1.067, *p* < 0.001), ES (HR = 1.016, 95% CI: 1.002–1.030, *p* = 0.023), ME (HR = 1.127, 95% CI: 1.054–1.204, *p* < 0.001), and RI (HR = 1.031, 95% CI: 1.013–1.048, *p* < 0.001) were independent predictors for EC patients.

### Survival-Associated AS-SF Network Constructing

**FIGURE 7:**
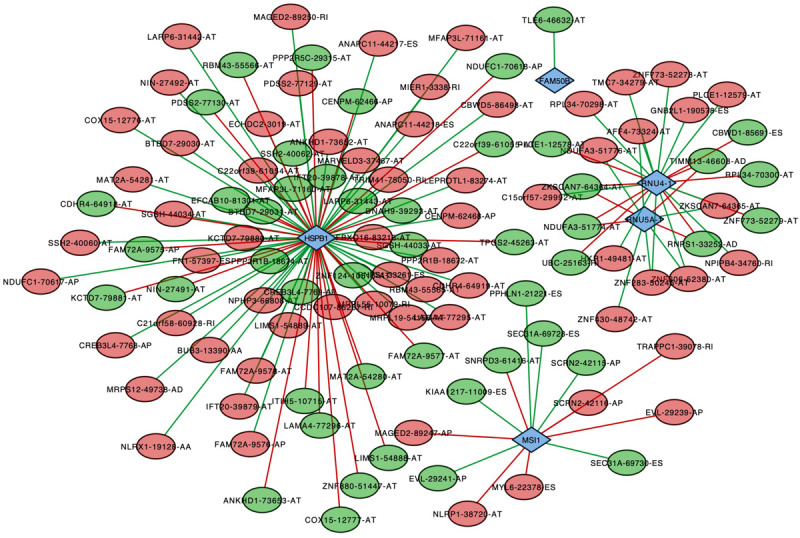
Splicing correlation network in endometrial cancer. Red dots represent the AS events whose PSI values are positively correlated with survival times. Green dots represent those whose PSI values are negatively correlated with survival times. Survival-associated factors are drawn in blue dots. The relationship between the PSI values of AS events and the expression of splicing factors were represented by a red line (positive correlation) and a green line (negative correlation).

To analyze the correlation between survival-associated AS events and splicing factors, a survival-associated AS-SF network was constructed based on the result of Pearson correlation test (Figure 7). The network contained 120 survival-associated AS events and 5 splicing factors (HSPB1, FAM50B, RNU4-1, RNU5A-1, and MSI1). We found that most high-risk prognostic AS events (red dots) were significantly negatively related to splicing factors (green lines). Conversely, most low-risk prognostic AS events (green dots) were significantly positively related to splicing factors (red lines).

## Discussion

Alternative splicing is one of the most important regulation mechanisms of the diversity of transcriptome and proteome. Some cancers can use AS to produce proteins that are conducive to the proliferation and invasion of cancer cells (Dargahi et al., 2014). Some AS events have been proven to be targets of prognosis and treatment (Pajares et al., 2007; Venables et al., 2008; Griffith et al., 2013). Several studies revealed that some cancer-associated AS variants, such as CD44 and VEGF (vascular endothelial growth factor) receptor, played an important role in cancer-targeted therapies (Heider et al., 2004; Sampson et al., 2008). Currently, the roles of AS events in the development of EC are still unknown.

In this study, several methods were used to screen prognosis-associated AS events and splicing factors based on the AS events data and clinical data of EC patients. We found that more than half of the genes undergo two or more AS events. It indicated that the splicing of genes was diverse and some of these AS events might produce disease-associated specific protein isoforms. According to the result from univariate COX analysis, 1826 AS events significantly associated with OS were selected (*p* < 0.05) and then LASSO algorithm was performed to develop prognostic signatures according to seven types of AS events (AA, AP, AT, AD, ME, ES, and RI).

With univariate Cox regression analysis and LASSO algorithm, survival-related AS events were selected and risk score models were developed by multivariate Cox regression analysis to estimate their prediction power. LASSO algorithm is a machine learning algorithm that can obviously improve the accuracy of prediction. As a result, all types of AS events were significantly associated with OS and prediction model of ES had satisfactory prediction accuracy (AUC = 0.866). Moreover, the risk score model of AA, AP, ES, ME, and RI were found as independent predictors for EC patients. Previous studies had developed predictor signatures related to the carcinogenesis and aggressiveness of EC based on other genomic features (Liu et al., 2019a, b). The ROC curves and KM curves certified that the classification of EC patients could be based on the survival-associated AS events prediction models. Our study further explored transcriptome changes in prognosis-related signatures, which was essential to understand how these signatures influenced the development of EC.

Functional enrichment analysis of genes that corresponded to survival-associated AS events was performed subsequently by Metascape. The top 3 terms included the PID–IL2–PI3K pathway, protein-containing complex localization, and vesicle targeting. The PID–IL2–PI3K pathway is involved in interleukin-2 (IL-2) signaling events that are associated with activated T lymphocytes mediated by PI3K, an important factor in regulating cellular metabolism and immune system function (Fruman et al., 2017). We also noticed that these genes also significantly clustered in some immune-related terms including adaptive immune system and B cell activation involved in immune response. These results meant that AS events corresponding to these genes might interfere with immune system and other biological processes affecting the development of EC.

Alternative splicing was the important reason of the diversity of mRNA, which were closely related to their own pre-mRNAs. Additionally, AS events in untranslated regions might lead to some abnormal events and cancer-related mRNA transcripts might activate the tumor suppressor, which influenced the carcinogenesis and aggressiveness of cancers (Chen and Weiss, 2015; Yang et al., 2018). In the PPI network, RPLP0, GRB2, MAPK, and NEK2 were the hub genes whose roles were already reported in EC (Takano et al., 2007; Artero-Castro et al., 2011; Wang et al., 2012; Gao and Zhang, 2015). RPLP0 had been reported as an important factor associated with the aggressiveness of EC (Artero-Castro et al., 2011). Shc–Grb2 complexes were one of the key proteins of the MEK/ERK pathway and played an important role in the proliferation, survival, and invasion in EC (Wang et al., 2012). MAPK served as a hub gene of various pathways, such as MAPK signaling pathway, the ErbB signaling pathway, and pathways involved in regulating the actin cytoskeleton, which mediated the cell proliferation and differentiation (Gao and Zhang, 2015). NEK2 had been found to be associated with the cell cycle of human endometrial stromal cells (Takano et al., 2007). In this study, we provided the in-depth mechanism of these factors in EC and novel methods for future clinical applications.

It is well known that various (and an abundance of) AS events were originated from limited splicing factors. In this study, we constructed a survival-associated AS-SF network to analyze the correlation between survival-associated AS events and splicing factors and show the larger regulated nodes. HSPB1 (heat shock protein B1), the most connected node, has been reported as having a significant role in EC (Korneeva et al., 2002). It was upregulated in EC and could inhibit induction of apoptosis. Based on this network, we could explore the possible mechanisms of HSPB1 in a deeper level.

## Conclusion

This study developed a prognostic prediction model based on the survival-related AS events and proved their predictive power. What we found in this study could provide a novel option for the prognostic prediction and treatment of EC patients. However, more experiments are still needed to explore the effects and mechanisms of dysregulated AS events and SFs in the development of EC.

## Data Availability Statement

Publicly available datasets were analyzed in this study. This data can be found here: TCGA-UCEC.

## Author Contributions

JW and PC carried out the study. PC analyzed and interpreted the data. PC and JH drafted the manuscript. HY and YL collected and analyzed the data. HY and SJ participated in the design and original draft writing. JW and XL coordinated the study, participated in the design, and reviewed the manuscript. All authors read and approved the final manuscript.

## Conflict of Interest

The authors declare that the research was conducted in the absence of any commercial or financial relationships that could be construed as a potential conflict of interest.

## Footnotes

1 https://xenabrowser.net

2 https://bioinformatics.mdanderson.org/TCGASpliceSeq/

3 http://metascape.org

## Supplementary Material

TABLE S1 1826 selected AS events of univariate Cox analysis.

TABLE S2 Selected key prognostic signatures of LASSO algorithm.
